# Publishing in Hematology Journals: A Scientometric and Economic Evaluation

**DOI:** 10.7759/cureus.12069

**Published:** 2020-12-14

**Authors:** Gokhan Tazegul, Unal Atas, Turgay Ulas, Tayfur Toptas, Ozan Salim

**Affiliations:** 1 Internal Medicine, Ankara Polatlı Duatepe State Hospital, Ankara, TUR; 2 Hematology, Akdeniz University, Antalya, TUR; 3 Hematology, Near East University, Nicosia, CYP; 4 Hematology, Marmara University, Istanbul, TUR

**Keywords:** hematology, health scientometrics, scientometrics, open access publishing, economics, open access

## Abstract

Introduction: Herein, we aimed to compare the scientometric data of hematology journals, and compare the publication models, especially the scientometric data of journals with all-open access (OA) and hybrid-OA publication models.

Methods: Data were obtained from Scimago Journal & Country Rank and Clarivate Analytics InCites websites. Fifty-four journals indexed in Science Citation Index (SCI) and SCI-Expanded were evaluated. Bibliometric data and impact factor (IF), scientific journal rank (SJR), eigenfactor score (ES), and Hirsch (h)-index of the journals were obtained. United States dollar (USD) was used as the requested article publishing charge (APC). Statistics Package for the Social Sciences (SPSS, IBM Corp., Armonk, NY) version 23.0 was used for data analysis.

Results: As a publication model, Hybrid-OA was the most common. One journal had subscription-only, and two journals had a free-OA model. Nine journals had a mandatory OA with the APC model and 42 journals used a hybrid model. The Median OA fee was 3400 USD. Hybrid-OA journals had a significantly higher median h-index (72 vs. 40, p=0.03) compared to all-OA journals. Other scientometric indexes were similar. When APCs were compared, all-OA journals were median 900 USD lower than hybrid-OA journals (2490 vs. 3400 USD, p=0.019).

Conclusion: There is a widespread use of the OA publication model in hematology journals. Although hybrid OA journals have higher h-index, other scientometric indexes are similar. All-OA journals are more economically feasible considering a lower median APC. Further scientometric studies for journals in the field of hematology, randomized to follow citation per publication according to the OA model would better shed light on the data in this area.

## Introduction

Medical journals are traditionally compared using quality indicators, such as scientometric indexes. These indexes apply varying methods to measure the number of citations published by articles in journals. These data are used for many different purposes, such as selecting journals for article submission, calculating the impact of publications, measuring the academic career of scientists, getting financial support from institutions, and purchasing subscriptions for libraries.

Impact factor (IF) is one of the scientometric indexes traditionally used to classify medical journals. IF is calculated by dividing the number of citations of articles published in a journal during specified time intervals by the number of articles published in that journal. Although IF is a popular scientometric index, it is also often criticized [1,2]. The Hirsch index (h), an alternative to IF, was originally developed to measure the scientific contributions of scientists and was later modified for journals. The h-index of a researcher or journal is defined as the h number of articles that have each been cited at least h times. However, this creates a positive bias toward more senior researchers [3,4]. Eigenfactor score (ES) and Scientific Journal Ranking scores (SJR) are computational models for measuring average prestige per paper, taking into account the formation of inter-citation networks like Google PageRank (i.e., considering the source of the citations) [5-7].

As a result of the widespread use of the internet, medical journals have shifted from the subscription publication model to the open-access (OA) publication model to meet the need of scientists to deliver their publications to more people. In the OA model, the journal acts as a service provider by evaluating the article/product for the scientist (peer review) and providing the most widespread distribution. Before OA, some medical journals requested submission, color figure, or page fees from authors, as well as subscription fees from libraries. However, with the OA model, the article processing/publishing charge (APC) has become one of the main revenue items in the financing of journals.

Over the years, some journals and publishing houses started publishing all papers as OA (all OA), while other journals started programs for optional OA publishing (hybrid OA). These developments began affecting the decisions of authors at many stages of the publishing process, from the evaluation of potential journals to the planning of funding requirements for their scientific studies. For example, some data indicate that OA journals receive more citations than subscription-based journals [8,9]. According to a study focusing on the amounts spent by German universities and research institutions on OA APCs, support for and financial contributions to OA publications have increased over the years, and APCs have also increased [10].

Therefore, with the advent of the internet age, there has been a paradigm shift in the selection of APC-charging journals in which researchers will publish [11]. Although several reports comparing the scientometric data of journals according to publication model have been published for different disciplines [12], no study on this subject has been conducted for the field of hematology. In this study, we aimed to (i) compare the scientometric data of journals publishing in the field of hematology, and (ii) compare the publication models of journals publishing in the field of hematology, especially the scientometric data of journals with all-OA and hybrid-OA publication models.

## Materials and methods

As this descriptive study uses publicly available and authorized data, it is exempt from ethics committee approval. The data used in this study were obtained from the Scimago Journal & Country Rank and Clarivate Analytics InCites websites between June and August 2020. The required permissions were provided by Clarivate Analytics and Scimago Lab and were added as a supplementary file. All other journal-related data used in this study were publicly available data collected from the websites of the journals and their publishers.

This study included journals that publish in the field of hematology and are indexed in the Science Citation Index (SCI) and SCI-Expanded within the Web of Science (99 journals in Scimago, 76 in Web of Science). The indexes of the journals were checked on the Web of Science InCites website, as well as on the website of each journal. To provide a robust analysis of journals publishing in the field of hematology, and to take into account the lack of reference studies in the literature, the following journals were excluded from the analysis: (i) journals that published fewer than 30 citable documents in the last three years or fewer than 10 in the last year (n = 4), (ii) multidisciplinary journals (such as journals that publish simultaneously in the fields of oncology, biochemistry, and immunology) that publish fewer than 25% of their original studies in the field of hematology (excluding special issues, such as conference proceedings or thematic issues; n=14), and (iii) invite-only journals (n = 4).

The data referring to total documents, citable documents, and total citations in the last three years, SJR, and h-index were obtained from the Scimago Journal & Country Rank database [13]. The ratio of articles printed under Creative Commons licensing with OA (gold open access) to total citable articles (% of citable OA documents), ES, and IF scientometric data were obtained from Web of Science InCites [14]. The quartiles of journals according to h-index, IF, ES, and SJR were recalculated using the Statistical Package for the Social Sciences (SPSS) software. The types of manuscripts published by the journals (original studies, reviews, case reports, and editorial articles) were categorized by examining the websites of the respective journals. The journals were consequently categorized into three types: (i) those that publish all types of papers, (ii) those that publish all types of papers except case reports, and (iii) those that publish only review papers. The OA publication models of the journals were categorized as (i) all OA (with APC), (ii) hybrid OA, (iii) free OA, and (iv) subscription only. The existence of a fee requested from the author for publication, whether the fee is requested before and/or after article acceptance, the amount of the fee, and the intended purpose of the fee were investigated using the data publicly available on the websites of the journals and publishers. The US dollar (USD) was used as the requested fee unit. A conversion rate of 1 USD = 0.85 EUR was used for two journals that did not specify USD in their pricing or did not specify their own conversion rate.

SPSS for Windows version 23.0 was used for data analysis. For descriptive data, continuous variables were expressed as a median (minimum-maximum) and categorical variables were expressed as numerical values and percentages. Chi-square testing was used to evaluate relationships between categorical data, Spearman's rho correlation was used for categorical correlations (ordinal by ordinal), and the Mann-Whitney U test was used for intergroup evaluations of continuous variables. A type-I error was accepted as 0.05.

## Results

Fifty-four journals that met the inclusion criteria were evaluated in this study. Their bibliographic information, scientometric data, and publication models were summarized in Table 1. Most of these journals published all types of scientific papers. The range of documents published in the last three years, the number of citable documents, and the total number of citations varied widely among journals. A hybrid-OA publication model was applied by most of the journals. Only one journal published under a subscription-only model, and two journals used a free-OA model. Across all journals, only 7.7% of all citable documents were published as OA. Fifteen of the journals charged a mandatory fee for publication. Four of these journals required a submission fee (of 20, 50, 75, and 75 USD, respectively) before manuscript acceptance. Five journals required a page charge fee after an article is accepted (25, 50, 75, 225, and 650 USD per page, respectively). Among the journals that apply an OA publication model, nine had a mandatory fee, and 42 journals used a hybrid model that offers optional participation in their OA model. The median OA fee was 3400 USD (Table 1).

**Table 1:** Bibliographic information, scientometric data, and publication models of the journals included in the study. OA: open-access publication model, IF: Impact factor, SJR: Scientific Journal Ranking score, ES: Eigenfactor score, all OA: publishing all papers as OA, hybrid OA: programs for optional OA publishing.

Types of published papers	
All types of papers	43 (79.6%)
No case reports	8 (14.8%)
Reviews only	3 (5.6%)
Bibliographic information	
Total documents (3 years)	423 (76-3077)
Citable documents (3 years)	344 (60-2168)
Total citations (3 years)	793 (26-23671)
Scientometric indexes	
h-index	66 (15-448)
IF	2.49 (0.52-17.54)
SJR	0.94 (0.174-5.41)
ES	0.004 (0.0001-0.205)
Publication model	
All OA	11 (20.4%)
Hybrid OA	42 (77.8%)
Subscription only	1 (1.8%)
Publication fee	
Mandatory	15 (27.8%)
Optional	37 (68.5%)
Free	2 (3.7%)
Fee timing	
Before	1 (1.9%)
After	48 (92.3%)
Both	3 (5.8%)
Open access participation	
All OA with APC	9 (17.0%)
Hybrid OA	42 (79.3%)
Free OA	2 (3.7%)

In this study, four different scientometric indexes were evaluated. The distributions of journal rankings into quartiles by these four indexes were compared using Spearman’s correlation coefficient (Table 2). A very strong positive correlation between SJR and IF, a moderate positive correlation between h-index and IF, and a strong positive correlation between all other pairs were observed. The lists of top 10 journals ranked by SJR and IF were similar, while the other scientometric indexes produced different rankings (Table 3).

**Table 2:** Spearman’s rho correlation coefficients of scientometric indexes. IF: Impact factor, SJR: Scientific Journal Ranking score, ES: Eigenfactor score.

	SJR	h-index	IF
h-index	0.613	-	-
IF	0.923	0.562	-
ES	0.789	0.778	0.752

**Table 3:** Side by side comparison of the top ten journals in different scientometric indexes. SJR: Scientific Journal Ranking score, IF: Impact factor, ES: Eigenfactor score.

SJR (reference)	IF	ES	h-index
#1	#1	#1	#1
#2	#4 (↑3)	#3 (↑2)	#3 (↑2)
#3	#2 (↓1)	#5 (↑2)	#13 (↑10)
#4	#3 (↓2)	#12 (↑8)	#12 (↑8)
#5	#6 (↑1)	#10 (↑5)	#10 (↑5)
#6	#5 (↓1)	#14 (↑8)	#5 (↓1)
#7	#7	#20 (↑13)	#17 (↑10)
#8	#9 (↑1)	#7 (↑1)	#15 (↑7)
#9	#12 (↑3)	#17 (↑12)	#18 (↑9)
#10	#15(↑5)	#13 (↑3)	#14 (↑4)

The percentages of citable OA documents were obtained from the Web of Science database. When compared with the scientometric indexes, the percentage of citable OA documents was moderately correlated with IF (R = 0.447, p = 0.001, Spearman's rho correlation) and weakly correlated with SJR (R = 0.375, p = 0.005, Spearman's rho correlation). There was no correlation with h-index and ES. For bibliographic data, the percentage of citable OA documents was not correlated with APC cost but was weakly correlated with the total number of citations (in the last three years; R = 0.284, p = 0.038, Spearman’s rho correlation).

The comparisons of bibliographic information, scientometric data, and publication models of journals that publish under all-OA and hybrid-OA models were presented in Table 4. The data regarding two journals that apply the free OA model and one journal with a subscription-only model were not presented. The median percentage of citable OA documents in journals publishing under hybrid-OA was 5.94%. Comparing the scientometric indexes of all-OA and hybrid-OA journals, only the h-index is significantly different: hybrid-OA journals had a significantly higher h-index (p = 0.03). However, when the journals in the first quartile of h-indexes were compared, both groups were found to be similar (p = 0.21). When APC fees were compared, the fees of all-OA journals were found to be a median of 900 USD lower than those of hybrid-OA journals (p = 0.019, Mann-Whitney U test).

**Table 4:** Comparison of bibliographic information and scientometric data of journals with all OA with APC and hybrid OA publishing models. OA: open-access publication model, all OA: publishing all papers as OA, APC: article processing/publishing charge (APC), hybrid OA: programs for optional OA publishing), IF: Impact factor, SJR: Scientific Journal Ranking score, ES: Eigenfactor score, USD: US dollar. *p<0.05, Mann-Whitney-U test.

	All OA with APC (n=9)	Hybrid OA (n=42)
Types of published papers		
All types of papers	9 (100%)	31 (73.8%)
No case reports	0 (0%)	8 (19%)
Reviews only	0 (0%)	3 (7.2%)
Bibliographic information		
Total documents (3 years)	398 (96-1109)	423 (76-1557)
Citable documents (3 years)	310 (92-654)	344 (60-1263)
Total citations (3 years)	656 (292-4747)	793 (26-7027)
Scientometric indexes		
h-index*	40 (22-136)	72 (15-185)
H-index (first quartile)	1 (11.1%)	13 (31%)
IF	3.5 (1.3-11)	2.4 (0.52-10.4)
IF (first quartile)	4 (44.4%)	10 (23.8%)
SJR	0.94 (0.44-3.23)	0.93 (0.17-4.19)
SJR (first quartile)	4 (44.4%)	10 (23.8%)
ES	0.003 (0.001-0.034)	0.004 (0.0001-0.048)
ES (first quartile)	3 (33.3%)	11 (26.2%)
Publication fee		
For submission	75 (1 journal)	50-75 (2 journals)
For page charges	Free	50-650 (4 journals)
APC* (USD)	2490 (700-4580)	3400 (1500-5000)

## Discussion

Scientometric data are used in decision-making in many areas of scientific research. However, none of the existing scientometric indexes are ideal. Much criticism has been leveled at the IF, a popular scientometric measurement, for reasons including its inability to evaluate between categories due to different citation patterns in different categories, its positive bias toward journals that publish reviews, its inclusion of self-citations, and its tendency to reduce the number of citable documents by including papers such as letters to the editor and clinical images [1,15]. Moreover, three out of four articles published in journals such as Science and Nature have lower citation rates than the IF of the journal itself, showing that the articles that increase the IF value of the journal constitute a very low percentage of the total published articles. For this reason, using the arithmetic mean of the citations may be misleading [2]. For example, a similar study in the field of sleep science observed that the journal with the highest IF was a review journal with an IF nearly twice as high as that of its closest competitor. However, when evaluated according to the h-index, the h-indexes of both journals are almost the same and they are both ranked in the first place [4]. Although IF and citation counts are frequently used in the ranking of journals, these values are commonly misused, and the negative aspects of their calculations are obvious. The h-index, an alternative scientometric measurement, can create a positive bias toward longer-running journals [3,4]. Other alternatives, such as the ES and SJR scores, have only recently started becoming widespread and have yet to be compared with other indexes. As interdisciplinary evaluations of these scientometric indexes are not possible due to biases, discipline-specific evaluations are obviously required. One of the aims of this study is to compare the scientometric data of journals that publish in the field of hematology.

In this study, we observed a very strong positive correlation between SJR and IF, a moderate positive correlation between the h-index and IF, and a strong positive correlation between all other pairs. Similarly, in another study comparing these four scientometric indexes in the field of environmental engineering, strong correlations were observed between these scientometric indexes [16]. In another study of journals that publish in the fields of radiology, nuclear medicine, and medical imaging, the R correlation coefficients between scientometric indexes were similar to those obtained in our study [17]. The lists of top 10 journals ranked by SJR and IF were similar, while the other scientometric indexes produced different rankings. When evaluated according to quartiles, the individual rankings changed, even if strong correlations had been observed. Similar results were seen in a study comparing journals publishing in the field of anatomy and morphology; there was no correlation between IF, ES, and SJR. The journal ranked in the first place according to IF was ranked twentieth according to ES and third according to SJR [7]. Therefore, although our results suggest that some indexes, especially SJR and IF, can be used interchangeably to evaluate journals publishing in the field of hematology, similar evaluations should also be performed for other scientific disciplines.

In the age of the internet, disseminating the products of scientific research - that is to say, scientific articles - as widely and quickly as possible has become a prevalent goal. The application of an OA publication model by medical journals has produced many results to that end. These developments have greatly changed individual and institutional behavior, from the way authors evaluate journals to how they determine the required funding for their scientific studies. For example, a study focusing on the amounts spent by German universities and research organizations on OA publication fees has shown increases not only in expenditures on OA publishing and APCs over the years but also in support for and financial contributions to OA publications [10].

In a study examining the factors evaluated by authors during journal selection, the most important factors were subject suitability, journal quality, and the speed of its review/publishing processes, and 60% of the participants stated that having whether the journal was OA is important [11]. The likely reason for this importance assigned to the OA publication model is that OA journals receive more citations than journals that publish under a subscription-only model. A study examining the five-year scientometric profile of 203 randomized controlled studies published in journals with and without OA showed that OA journals receive 1.3 times more citations than those without OA [8]. OA status did not affect citations in the field of ophthalmology [18]; in psychiatric journals, it was one of three main factors that positively influenced the number of citations, along with the study design being included in the paper title and having a structured abstract [9].

In this study, we have shown that most journals in the field of hematology apply the hybrid-OA model and the median of citable OA documents in these journals is very low (5.94%). An all-OA model is applied by 11 journals, nine of which charge an APC. No significant difference was found in scientometric indexes in favor of the all-OA publication model. Therefore, these results show that scientometric indexes should be evaluated specifically for each discipline.

This paradigm shift in the publishing model also affected the economic aspect of scientific publications. Both the number of OA journals and APCs tend to increase over the years [10]. In an article that previously discussed author plans for APC in the OA publication model, it was observed that the authors used institutional funds or research grants for journals with high APC as resources, and personal fund payments were preferred for low APCs. At the same time, personal fund payments are preferred in low-income countries [11].

Chua et al. observed that IF does not correlate with the number of citations in OA journals, but the number of citations and the IF of non-OA journals is moderately correlated. Therefore, considering that APC increases with increasing IF, it may make sense to submit articles for more citations to OA journals, but it may not be reasonable to pay more by choosing journals with higher IF [8]. In this study, we observed that 15 journals demand a fee from the authors for any reason, and nine of these journals demand an APC under an all-OA model. When all-OA and hybrid-OA journals were compared, all-OA journals were shown to be a median of 900 USD cheaper. Considering that all-OA journals are similar to hybrid-OA journals in terms of scientometric indexes, it may be economically reasonable to prefer all-OA journals for publishing in the field of hematology.

Our study has certain limitations. First, since our study was planned for the data available on a per-journal basis rather than on a per-paper basis, the inferences made about OA were determined for the journal-based data. Although similar results are found in paper-based studies on OA, a separate evaluation of OA articles in the field of hematology may yield different results. The second and most important limitation is that the scientometric data, APCs, and bibliographic data are taken from databases. In particular, APCs are subject to waivers or discounts on papers from low-income countries or contracted institutions. Furthermore, scientometric data can change from database to database. Therefore, this situation could not be taken into account in these analyses.

## Conclusions

When evaluating a journal, a paper, or a scientist using scientometrics, one should consider the advantages and disadvantages of scientometric data, as well as the extent to which they correlate with each other in the respective field of science. There is a widespread use of the OA publication model in hematology journals, however, in hybrid OA journals, a very low percentage of papers are published as OA. All OA and hybrid OA journals are similar regarding scientometric indexes, apart from a higher h-index in hybrid-OA journals. Journals that publish as all OA are more economically feasible. This journal-based study is the first to document the publication profile of journals in hematology. Further scientometric studies for journals in the field of hematology, randomized to follow citation per publication according to OA models would better shed light on the data in this area.
